# Low rate of infectious mortality omitting fluoroquinolone prophylaxis in high-risk hematological patients, a single centre experience

**DOI:** 10.3389/fmicb.2025.1632055

**Published:** 2025-10-17

**Authors:** Adele Santoni, Margherita Malchiodi, Elisabetta Zappone, Alessandra Cartocci, Anna Sicuranza, Paola Pacelli, Corrado Zuanelli Brambilla, Marzia Defina, Mario Tumbarello, Monica Bocchia

**Affiliations:** ^1^Hematology Unit, Azienda Ospedaliera Universitaria Senese, University of Siena, Siena, Italy; ^2^Department of Medical Science, Surgery and Neuroscience, University of Siena, Siena, Italy; ^3^Infectious Diseases Unit, Azienda Ospedaliera Universitaria Senese, University of Siena, Siena, Italy

**Keywords:** febrile neutropenia, fluoroquilone, bloodstream infection (BSI), hematological malignancies, prophylaxis, acute myeloic leukemia (AML), acute lymphobastic leukemia, non-Hodgkin lymphoma

## Abstract

**Introduction:**

In hematological patients treated with intensive chemotherapy (CHT), febrile neutropenia (FN) is the primary cause of non-relapse mortality (NRM) due to infections that occur during prolonged neutropenia. Fluoroquinolone (FQ) prophylaxis is still recommended by several guidelines for neutropenic patients because it helps reduce bacterial infection rates and fever episodes, although it does not affect infection-related mortality (IRM). However, in the era of multi-drug resistance (MDR), the use of FQs should be evaluated carefully.

**Methods:**

We present a retrospective, single-center study based on real-life data that includes 512 intensive chemotherapy treatments and the occurrence of prolonged neutropenia in 236 high-risk (HR) hematological patients treated without FQ prophylaxis.

**Results:**

In the entire cohort, we recorded FN in 80.5% of the cases. Among these, 33.7% were attributed to fevers of unknown origin, 45.4% were associated with bloodstream infections (BSIs), 9.0% involved bacterial organ infections, 13.6% were due to fungal infections, and 3.4% were linked to viral infections. Septic shock was observed in 7.6% of the patients. Although we documented a high infection rate, the IRM and overall mortality rates were 3.0% (7/236) and 9.3% (22/236), respectively. These rates are comparable to those found in settings where FQ prophylaxis is used.

**Conclusion:**

Although our cohort was small, our results advocate for the exclusion of FQ prophylaxis in HR hematological patients, without increasing the IRM rate and addressing the risk of life-threatening MDR infections. While we believe it is mandatory to have an efficient protocol for the prompt treatment of FN, our data should encourage hematologists to limit the use of FQ prophylaxis.

## Introduction

In high-risk (HR) hematological patients, febrile neutropenia (FN) is a frequent emergency that can be fatal, especially during induction chemotherapy (CHT) and high-dose (HD) consolidation CHT, representing the leading cause of non-relapse mortality (NRM) (Garcia-Vidal et al., 2019; Owattanapanich and Chayakulkeeree, 2019; Singh et al., 2023; Douglas et al., 2022). According to the Infectious Diseases Society of America (IDSA) guidelines and National Comprehensive Cancer Network (NCCN) guidelines (Taplitz et al., 2018), patients receiving high-dose CHT are at a very high risk for infections due to prolonged aplasia periods (absolute neutrophil count (ANC) < 500/mmc and neutropenia > 7 days) (Freifeld et al., 2011; Bow, 2013; Bodey et al., 1966). This risk is attributed to both myelosuppressive CHT consequences and disease effects on the immune system (Torres-Flores et al., 2020). Furthermore, the use of central venous catheters (CVCs) and peripherally inserted central catheters (PICCs) can create an entry point for pathogens, causing the disruption of the skin barrier (Bennett et al., 2020). Finally, bloodstream infections (BSIs) could also be caused by the disruption of the gastrointestinal barrier. Indeed, CHT affects intestinal epithelial cells by increasing mucosal permeability, which can lead to infectious complications mediated by the gut microbial community (Girmenia and Gentile, 1999). Consequently, hematological patients can frequently develop BSIs and organ infections such as enterocolitis, pneumonia, and urinary tract infections, increasing their morbidity and mortality rates (Torres-Flores et al., 2020). Although Gram-positive BSIs are on the rise, Gram-negative BSIs are currently the most frequent (Carvalho et al., 2020; Trecarichi et al., 2015). In some cases, these infections are caused by multidrug-resistant (MDR) Gram-negative bacteria (Trecarichi et al., 2023; Trecarichi and Tumbarello, 2014), particularly extended-spectrum beta-lactamase (ESBL)-producing and carbapenem-resistant Enterobacteriaceae (CRE) (Tumbarello and Raffaelli, 2021).

BSIs sustained by Gram-negative bacteria and septic shock are extremely difficult to manage, and they can rapidly lead to multi-organ failure and death, especially when MDR strains are involved (Gershkovich et al., 2019; Micozzi et al., 2017; Pagano and Caira, 2014; Sheikh et al., 2022). In this scenario, anti-bacterial prophylaxis and prompt empiric treatment are essential for managing HR hematological patients with prolonged neutropenia to reduce morbidity and mortality (Owattanapanich and Chayakulkeeree, 2019; Paul et al., 2010; Egan et al., 2019; Bucaneve et al., 2005). Since 2007, major international guidelines have continued to recommend fluoroquinolone (FQ) prophylaxis for HR hematological patients with prolonged neutropenia (>7 days) in order to reduce both neutropenic fever and systemic Gram-negative bacterial infections and consequent infection-related mortality (IRM) (Taplitz et al., 2018; Bucaneve et al., 2007; Phillips et al., 2012; Christopeit et al., 2020; Tallman et al., 2019). However, recent studies confirm that while FQ prophylaxis reduces BSI rates and FN episodes, it does not impact IRM (Trecarichi et al., 2023). This evidence, together with the increasing global prevalence of FQ-resistant strains and the risk of selecting MDR pathogens, has sparked discussions about the real benefits of this prophylaxis. In addition, the European Conference on Information Literacy (ECIL), since 2017, recommends using FQ prophylaxis based on local epidemiology (Hakki et al., 2019; Mikulska et al., 2018; European Centre for Disease Prevention and Control, 2019; Baden et al., 2016). Furthermore, recent retrospective studies have shown that omitting FQ prophylaxis in acute myeloid leukemia (AML) patients was not associated with higher induction IRM (Urbino et al., 2023; Santoni et al., 2023; Cernan et al., 2023). However, the use of antibiotics increases the risk of Clostridioides difficile infections, (Hensgens et al., 2012) induces gut microbiome dysbiosis (Daoud-Asfour et al., 2022; Leardini et al., 2022), and may promote invasive fungal infections (IFIs) (Kofteridis et al., 2017).

Despite these findings, some reports still recommend the use of fluoroquinolones (Caro et al., 2022; Maakaron et al., 2020; Kern et al., 2018), and FQ prophylaxis remains a grade A recommendation in several guidelines. However, due to their side effects, the Food and Drug Administration has imposed restrictions on their use (Taplitz et al., 2018; Freifeld et al., 2011; Barberán et al., 2023). Notably, the ECIL-8 group does not recommend antibacterial prophylaxis in pediatric hematological patients, considering that it reduces BSIs without providing a survival benefit (Lehrnbecher et al., 2021). In Italy, antibiotic resistance is higher compared to other European countries, and the Health Department is strongly focusing on prevention, monitoring, and reducing antibiotic use to contain infections (Piano Nazionale di Contrasto dell’Antimicrobico-Resistenza (PNCAR), 2017-2020) A recent multicenter Italian study documented a correlation between FQ prophylaxis and cephalosporin resistance in E.coli that caused BSIs in hematological patients (Trecarichi et al., 2019). This finding reinforces a local, epidemiology-based approach to prophylaxis (Mikulska et al., 2018) and has prompted many Italian institutions to limit the use of FQ (Piano Nazionale di Contrasto dell’Antimicrobico-Resistenza (PNCAR), 2017-2020). In line with these principles, since 2014, we have stopped administering FQ prophylaxis in neutropenic HR hematological patients.

This current study aimed to retrospectively assess the rates of bacterial infections, BSIs, fevers of unknown origin (FUO), and IRM in neutropenic patients with hematological malignancies who received HD CHT without FQ prophylaxis, while implementing an efficient protocol for the prompt treatment of these patients during their hospital stay.

## Patients and methods

We retrospectively analyzed the outcomes of 236 consecutive adult patients (age ≥ 18 years) treated at the Hematology Unit of Siena University Hospital (Italy) for acute myeloid leukemia (AML), excluding non-M3 AML subtypes, acute lymphoblastic leukemia (ALL), or aggressive non-Hodgkin lymphoma (NHL) from January 2015 to December 2022.

Patients’ charts were manually reviewed to collect data, including demographics, disease characteristics, treatment, length of hospital stay, duration of neutropenia, infections that occurred, and their outcomes. All data were entered into a database.

All data were handled in accordance with local regulations.

The patients shared two-bed rooms and underwent a total of 512 HD CHT treatments (288 induction and 224 consolidation cycles), resulting in neutropenia (ANC < 500/mmc) lasting 7 days or longer, without receiving FQ prophylaxis. CHT was administered using a CVC or a PICC.

The patients received high-intensity treatment based on their specific diagnoses, as detailed in the Supplementary material.

Upon admission, the patients underwent a chest X-ray to exclude pulmonary infections or pleural effusion, and starting from March 2020, they were tested for SARS-CoV-2.

Anti-*Pneumocystis jirovecii* prophylaxis with trimetoprim/sulfamethoxazole 800/160 mg was given twice weekly, and antifungal prophylaxis was administered to all patients until ANC < 500/mmc. The antifungal prophylaxis consisted of posaconazole (suspension or tablets) 300 mg daily or intravenous liposomal amphotericin B 50 mg every 2 days when administered concomitantly with vinca alkaloid. Antiviral prophylaxis with acyclovir 400 mg twice daily was provided only to patients with ALL and NHL.

Granulocyte colony-stimulating factor (G-CSF) was administered to all neutropenic patients until neutropenia recovery (ANC > 500/mmc), as indicated in the protocols used.

At hospital admission, all patients underwent bacterial colonization surveillance through culture and molecular analysis of rectal swabs to detect possible ESBL or CRE colonization, utilizing the multiplex PCR method. In cases of a positive molecular test and negative culture, two rectal swab cultures were repeated after 24 and 48 h. Patients were considered negative if all three subsequent culture tests were negative. In cases where both molecular and cultural tests were positive, functional contact isolation was implemented for all ESBL+ colonized patients, while single-room isolation was implemented for the few CRE + colonized patients. All patients underwent weekly rectal swab culture screening.

In cases of fever of ≥38°C in two consecutive surveys and/or shivering, or conversely hypothermia with a body temperature of <35.5°C, our specific protocol mandates the following sequence: collection of two sets of blood cultures both from a peripheral vein and each central venous line for aerobic and anaerobic bacteria, high rapid fluid administration, antibiotic administration within 1 h from the onset of fever, and close monitoring of vital signs. In addition, serum lactate, procalcitonin, and galactomannan were promptly measured; neutropenic fever was treated with first-line empiric antibiotic therapy, including either ceftazidime or piperacillin/tazobactam, both combined with amikacin. The ESBL+ patients received standard first-line therapy, while in the CRE + colonized patients, the first-line antibiotic therapy was based on the sensitivity/resistance of the colonizing bacteria. If the colonizing bacteria were not isolated in blood cultures, de-escalation was attempted while still maintaining broad-spectrum therapy. In cases of MDR bacteria isolation, antibiotic therapy was adjusted according to the antibiogram, following the EUCAST clinical breakpoint guidelines.

In cases of persistent fever lasting 48–72 h, second-line antibiotic therapy was administered by adding vancomycin and eventually followed by third-line antibiotic therapy with Meropenem, maintaining vancomycin and discontinuing ceftazidime and amikacin. Empirical antifungal therapy with liposomal Amphotericin B at a dosage of 3–6 mg/Kg/day was started in cases of clinical, radiological, or laboratory-suspected fungal infection. If indicated, a high-resolution CT (HRTC) scan of the chest was performed, and in the presence of pulmonary signs of infection, a bronchoalveolar lavage (BAL) for extended microbiological investigation was obtained.

### Definitions

Neutropenia was defined as an ANC of <500/mmc, while severe neutropenia was defined as an ANC of <100/mmc (Klastersky et al., 2016). It was considered prolonged if its duration was ≥7 days.

BSI was defined as the detection of a bacterial or fungal pathogen in the blood samples (Wang et al., 2024).

Viral infections were defined as positive based on the detection of a positive specific PCR test and clinical or radiological evidence.

Fungal infections were defined as positive based on the detection of a positive *Aspergillus* galactomannan antigen, a positive specific PCR test, or radiological evidence.

FN without evidence of BSI or organ-specific infection (i.e., pneumonia or urinary or gastrointestinal tract infections, with or without microbiological isolation) was defined as FUO (Weir Restrepo et al., 1999).

All hypotensive conditions requiring the use of vasopressors to maintain a mean arterial pressure (MAP) of >65 mmHg despite volume filling were defined as shock (Singer et al., 2016).

IRM was defined as infection-related death within 60 days of hospital admission, and overall mortality was defined as death from any cause within 60 days of hospital admission.

### Statistical analysis

Descriptive statistics were carried out. The median and interquartile range (IQR) were calculated for non-normally distributed quantitative variables, and the mean and standard deviation were calculated for normally distributed variables. Absolute frequencies and percentages were estimated for qualitative variables. The Mann–Whitney *U* test or Student’s *t*-test was used to evaluate differences in quantitative variables (age, days of hospitalization, days of neutropenia, and days of severe neutropenia) between the patients with and without fever, FUO, and BSI. Fisher’s exact test was used to assess the association between qualitative variables (sex, fever, FUO, organ infection, rectal swab colonization, ESBL, and CRE colonization) and the patients with and without fever, FUO, and BSI. All analyses were conducted by stratifying patients into AML, ALL, NHL, and the total lymphoid population (NHL + ALL). An ANOVA test, Kruskal–Wallis test, or chi-squared test was performed to compare the characteristics of AML, ALL, and NHL. If these tests were significant, multiple *t*-tests, Mann–Whitney U tests, or Fisher’s exact tests were performed, with false discovery rate correction applied. The Kaplan–Meier method was used to plot overall survival (OS) and infection-free survival (IFS). A *p*-value of <0.05 was considered statistically significant. All the analyses were carried out with R version 4.3.2.

## Results

### Patient population

From January 2015 to December 2022, a total of 236 HR hematological patients were included in our study, of whom 54.6% were male and 45.4% female, with a median age of 59 years [18–88]. In our population, 70% of the patients had AML, 18.6% had ALL, and 11.4% NHL. They received a total of 512 CHT treatments, including 288 induction and 224 consolidation treatments.

The median duration of neutropenia (ANC < 500/mmc) was 15 days [7–63] in the entire population, and the median duration of severe neutropenia (ANC < 100/mmc) was 12 days [1–54]. The patients’ characteristics are summarized in Table 1. Comparing the three groups, the differences in the duration of severe neutropenia and median hospitalization between the AML group and the combined lymphoid group (ALL+NHL) were statistically significant (*p* < 0.001).

**Table 1:** Patients’ characteristics.

Patients’ characteristics	Total	AML^1^	ALL^2^	NHL^3^
Pts^4^	236	165	44	27
Median age (years)	59.0 (18–88)	58.6 (18–88)	46.5 (18–75)	56.4 (20–84)
Sex				
Male (%)	129/236 (54.6)	84/165 (50.9)	24/44 (54.5)	21/27 (77.8)
Female (%)	107/236 (45.4)	81/165 (49.1)	20/44 (45.5)	6/27 (22.2)
Cht^5^ treatment	512	379	90	43
Induction (%)	288/512 (56.3)	210/379 (55.4)	51/90 (56.7)	27/43 (62.8)
Consolidation (%)	224/512 (43.7)	169/379 (44.6)	39/90 (43.3)	16/43 (37.2)
Hospitalization (days)	23 (8–80)	24 (21–27)	20 (18–27)	20 (17–23)
Neutropenia (days)				
ANC^6^ < 500/mmc	15 (7–63)	17 (13–22)	12 (8–20)	10 (8–12)
ANC < 100/mmc	12 (1–54)	13 (9.5–18)	8 (5–12)	6 (5.5–9)
Fever (%)	412/512 (80.5)	318/379 (83.9)	61/90 (67.8)	33/43 (76.7)
FUO^7^ (%)	139/412 (33.7)	103/318 (32.4)	26/61 (42.6)	10/33 (30.3)
BSIs^8^ (%)	187/412 (45.4)	143/318 (45.0)	27/61 (44.3)	17/33 (51.5)
Gram-positive (%)	65/187 (34.8)	53/143 (37.1)	6/27 (22.2)	6/17 (35.3)
Gram-negative (%)	114/187 (61.0)	83/143 (58.0)	20/27 (74.1)	11/17 (64.7)
Both (%)	8/187 (4.2)	7/143 (4.9)	1/27 (3.7)	0/17 (0.0)
Bacterial organ infections (%)	46/412 (11.2)	33/318 (10.4)	8/61 (13.1)	5/33 (15.2)
Fungal Infections	56/412 (13.6)	41/318 (12.9)	8/61 (13.1)	7/33 (21.2)
Viral infections (%)	14/412 (3.4)	10/318 (3.1)	2/61 (3.3)	2/33 (6.1)
Shock (%)	39/512 (7.6)	27/379 (7.1)	7/90 (7.8)	5/43 (11.6)
Rectal swab (%)	118/512 (23.0)	84/379 (22.2)	28/90 (31.1)	6/43 (14.0)
ESBL^9^ (%)	96/118 (81.4)	68/84 (81.0)	23/28 (82.1)	5/6 (83.3)
CRE^10^ (%)	20/118 (16.9)	15/84 (18.0)	4/28 (14.3)	1/6 (16.7)
Both (%)	2/118 (1.7)	1/84 (1.0)	1/28 (3.6)	0/6 (0.0)
30-day mortality (%)	22/236 (9.3)	17/165 (10.3)	2/90 (2.2)	3/43 (7.0)
Infectious related (%)	7/236 (3.0)	6/165 (3.6)	1/90 (1.1)	0/43 (0.0)

^1^Acute Myeloid Leukemia. ^2^Acute Lymphoblastic Leukemia. ^3^Non Hodgkin Lymphoma. ^4^Patients. ^5^Chemotherapy. ^6^Absolute Neutrophil Count. ^7^Fever of Unknown Origin. ^8^Bloodstream Infections. ^9^Extended Spectrum Beta-Lactamases. ^10^Carbapenem-Rresistant Enterobacterales.

### Febrile neutropenia and microbiology

A total of 412 (80.5%) FN episodes were documented among the 512 HD CHT treatments. The incidence was significantly higher (*p* = 0.002) in the AML population (318/379, 83.9%) compared to the ALL (61/90, 67.8%) and the NHL populations (33/43, 76.7%). FN episodes correlated with the duration of both neutropenia (ANC < 500/mmc) and severe neutropenia (ANC < 100/mmc) in the AML group (*p* < 0.001 for both), while they correlated only with severe neutropenia in the ALL group (*p* = 0.026) and with the length of hospitalization but not with neutropenia in the NHL group (*p* = 0.019).

**Figure 1:**
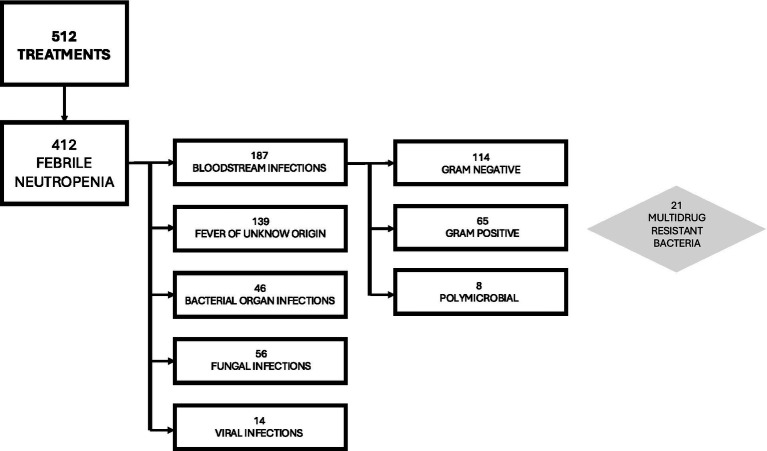
Flow chart about febrile neutropenia and microbiology of the cohort study.

Analyzing FN episodes, we documented 139/412 (33.7%) as FUO, 187/412 (45.4%) as BSIs, 46/412 (11.2%) as bacterial organ infections, 56/412 (13.6%) as fungal infections, and 14/412 (3.4%) as viral infections, as detailed in Table 1 and Figure 1.

Considering FUO, there was a statistically significant correlation between FUO and longer hospitalization in the AML group (*p* = 0.039) and the ALL group (*p* = 0.035) but not in the NHL group (*p* = 0.288). In the ALL group, FUO also correlated with longer durations of neutropenia (ANC < 500/mmc, *p* = 0.027) and severe neutropenia (ANC < 100/mmc, *p* = 0.005).

Among the BSIs, 65/187 (34.8%) were caused by Gram-positive bacteria, 114/187 (61%) by Gram-negative bacteria, and 8/187 (4.2%) were polymicrobial BSIs, caused by both Gram-negative and Gram-positive bacteria. BSI was significantly associated with longer durations of neutropenia (ANC < 500/mmc, *p* = 0.005) and severe neutropenia (ANC < 100/mmc, *p* = 0.035) only in the ALL group, while in the AML group, BSI correlated with the length of hospitalization (*p* = 0.003) but not with neutropenia or severe neutropenia (*p* = 0.634 and *p* = 0.452, respectively).

**Figure 2:**
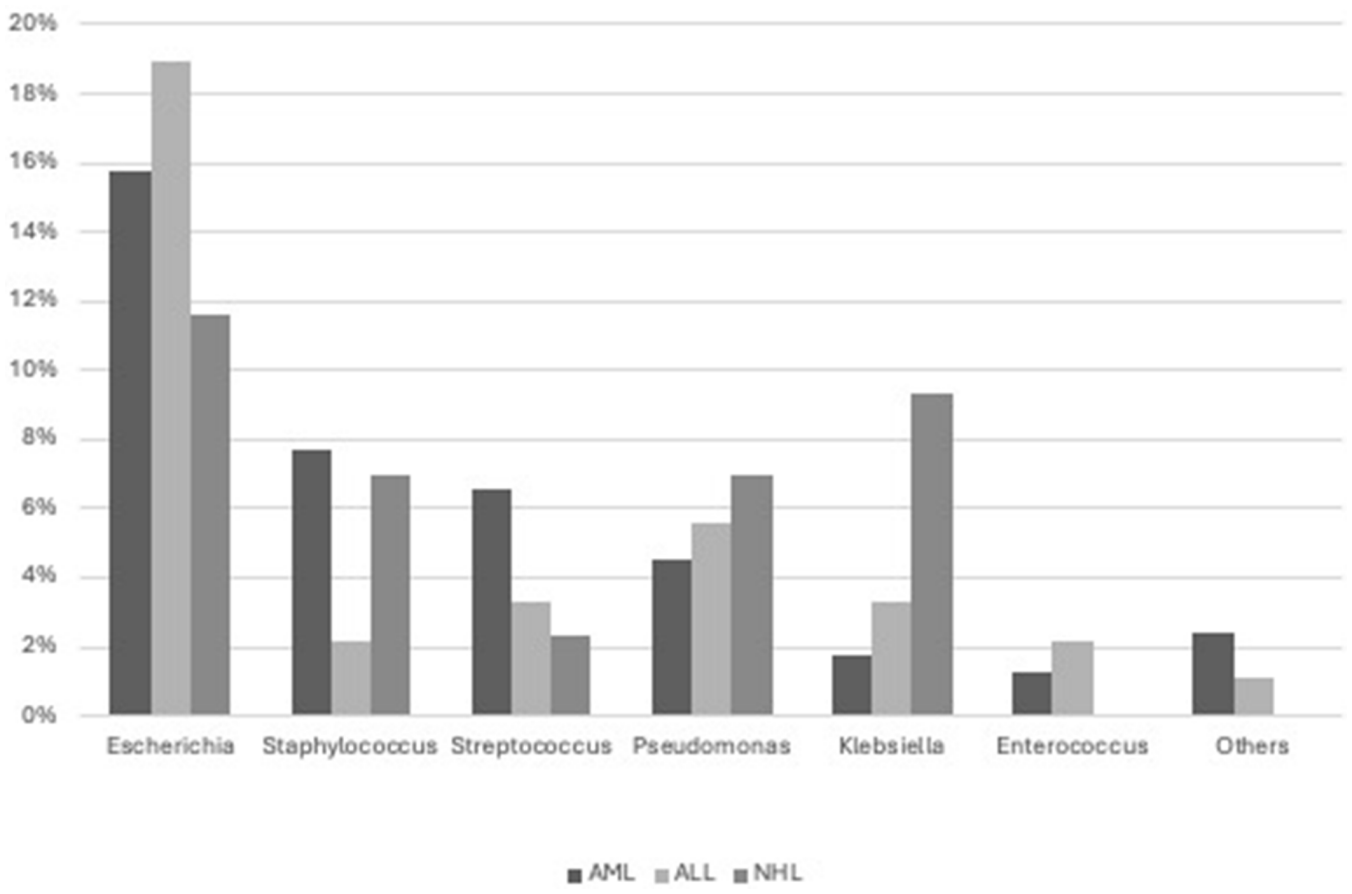
Microorganisms sustained sepsis.

BSI was predominantly caused by *Escherichia coli* (79, 12 ESBL+), *Staphylococci* (33, 1/33 S.aureus MRSA), *Streptococci* (European Centre for Disease Prevention and Control, 2019), *Pseudomonas aeruginosa* (25, 2 MDR), *Klebsiella pneumoniae* (14, 3 ESBL+, 2 CRE), and *Enterococcus faecium* (8, 1 vancomycin-resistant). Less commonly, it was caused by other bacteria such as *Citrobacter* (Owattanapanich and Chayakulkeeree, 2019), *Rhodococcus* (2), *Corynebacterium* (1), *Bacteroides* (1), *Moraxella* (1), *Serratia* (1), *Campylobacter* (1), *Suttonella* (1), and *Achromobacter* (1) (Figure 2).

Specifically, 21/187 (11,2%) BSIs were caused by MDR bacteria.

Furthermore, in our population, we documented 5/512 (1%) microbiologically confirmed colonized CVCs.

Septic shock occurred in 39/512 cases (7.6%), with similar incidences across the three groups: 27/379 (7.1%) in AML, 7/90 (7.8%) in ALL, and 5/43 (11.6%) in NHL, with no statistically significant differences. Only one patient was transferred to the intensive care unit after developing acute renal failure and multiorgan failure, which resulted in death. He also developed pneumonia without any microbiological findings in the blood culture or BAL and without radiological evidence of possible invasive fungal infection (IFI).

Regarding bacterial organ infections, we recorded 46 cases of the 512 treatments (Table 2). Specifically, 33 of 379 patients with AML (8.7%) experienced bacterial organ infections, which were significantly correlated with longer hospitalization (*p* < 0.001) and documented pneumonia (*p* < 0.001). In contrast, among the patients with ALL and NHL, bacterial organ infections were detected in 8 of 90 (8.9%) and 5 of 43 (11.6%) patients, respectively, without any significant correlation.

**Table 2:** Organ infections.

Type	Total cycles	AML^1^	ALL^2^	NHL^3^
Infections	46/512 (8,9%)	33/379 (8,7%)	8/90 (8,9%)	5/43 (11,6%)
Enteritis	14/512 (2,7%)	9/379 (2,4%)	2/90 (2,2%)	3/43 (7%)
Cl.difficile	8/512(1,6%)	7/379 (1,8%)	1/90 (1,1%)	0/43 (0%)
Respiratory infections	25/512 (4,9%)	20/379 (5,3%)	4/90 (4,4%)	1/43 (2,3%)
Pneumonia	24/512 (4,7%)	20/379 (5,3%)	3/90 (3,3%)	1/43 (2,3%)
Urinary infections	11/512 (2,1%)	7/379 (1,8%)	3/90 (3,3%)	1/43 (2,3%)
Skin infections	6/512 (1,2%)	6/379 (1,6%)	0/90 (0%)	0/43 (0%)

^1^AML: acute myeloid leukemia. ^2^ALL: acute lymphoblastic leukemia. ^3^NHL: non-hodgkin lymphoma.

### MDR bacteria colonization

MDR bacteria colonization, assessed via rectal swabs, was also analyzed. It was found in 118/512 (23%) cases, including 96/512 (18.8%) with an ESBL-producing organism, 20/512 (3.9%) with CRE, and two (0.4%) with double colonization. ESBL colonization was found in 69/379 (18.2%) patients with AML, 24/90 (26.7%) patients with ALL, and 5/43 (14%) patients with NHL, while CRE colonization was detected in 16/379 (4.2%) patients with AML, 5/90 (5.6%) patients with ALL, and 1/43 (2.3%) patients with NHL.

Colonization by ESBL+ or CRE was not statistically different across the three disease groups (*p* = 0.079 and *p* = 0.684, respectively). In addition, within each subgroup, colonization was not associated with a statistically significant risk of BSI caused by the same pathogen (*p* > 0.05). Moreover, we did not find any correlation between ESBL and CRE colonization and mortality (*p* > 0.05).

### Mortality

A total of 22/236 patients died within 60 days of hospital admission, with a 60-day OM of 9.3%. Regarding 60-day IRM, only 7/236 (3%) patients died due to infections during induction CHT: 2/236 (0.8%) patients died from pulmonary aspergillosis, 3/236 (1.3%) patients from acute respiratory distress caused by pneumonia, and finally 2/236 (0.8%) patients died from septic shock. No patients died during consolidation CHT (*p* < 0.001).

### Induction versus consolidation treatments

Data regarding induction and consolidation treatments are summarized in Table 3.

**Table 3:** Induction versus consolidation treatments.

		Induction	Consolidation	*p*
Chemotherapy		288/512 (56.3%)	224/512 (43.7%)	
AML^1^		210/288 (72.9%)	169/224 (75.5%)	
	ANC^4^ < 500/mmc (days)	20 (15–24)	14 (11–17)	**<0.001**
	ANC < 100/mmc (days)	15 (12–20)	11 (8–14)	**<0.001**
	Hospitalization (days)	25 (22–28)	23 (21–26)	**0.003**
	Fever	188/210 (89.5%)	129/169 (76.3%)	**0.001**
	FUO^5^	65/188 (34.6%)	38/129 (29.5%)	0.405
	BSIs^6^	71/188 (37.8%)	71/129 (55.0%)	**0.003**
ALL^2^		51/288 (17.7%)	39/224 (17.4%)	
	ANC < 500/mmc (days)	17.5 (12–22)	8 (8–11)	**<0.001**
	ANC < 100/mmc (days)	12 (8–17.75)	6 (5–7)	**<0.001**
	Hospitalization (days)	26 (21.50–30.50)	18 (16–19)	**<0.001**
	Fever	39/51 (76.5%)	22/39 (56.4%)	*p* = 0.073
	FUO	13/39 (33.3%)	13/22 (59.1%)	*p* = 0.092
	BSIs	19/39 (48.7%)	8/22 (36.4%)	*p* = 0.506
NHL^3^		27/288 (9.4%)	16/224 (7.1%)	
	ANC < 500/mmc (days)	11 (9–14)	8 (7.75–9.25)	**0.002**
	ANC < 100/mmc (days)	8 (6–10.5)	6 (5–6)	**0.012**
	Hospitalization (days)	23 (19–25)	18 (17–19.5)	**0.002**
	Fever	22/27 (81.5%)	11/16 (68.8%)	*p* = 0.561
	FUO	6/22 (27.3%)	4/11 (36.4%)	*p* = 0.893
	BSIs	5/11 (45.5%)	12/22 (54.5%)	*p* = 0.902

^1^Acute Myeloid Leukemia. ^2^Acute Lymphoblastic Leukemia. ^3^Non Hodgkin Lymphoma. ^4^Absolute Neutrophil Count. ^5^Fever of Unknown Origin. ^6^Bloodstream Infections.

In all groups, both neutropenia and, consequently, hospitalization were significantly longer during induction compared to consolidation treatment, with statistical significance.

Analyzing fever episodes, we found a higher incidence of fever during induction treatments, with a statistical significance in the AML population.

## Discussion

FQ prophylaxis in HR hematological neutropenic patients remains a challenge for hematologists, balancing evolving recommendations with controversial real-world data. Since 2014, due to the overall rising antibiotic resistance in Italy, the subsequent implementation of strong antimicrobial resistant stewardship programs, and local epidemiological data showing high FQ resistance, we chose to omit FQ prophylaxis in this HR population by implementing a twofold strategy: routine surveillance cultures using rectal swabs and a prompt, broad-spectrum empiric antibiotic approach tailored to the patient’s colonization status for the management of febrile neutropenia.

In this single-center retrospective study, we analyzed a large cohort of 236 HR hematological patients with more than 7 days of neutropenia, reporting the infection rate in relation to our local bacterial epidemiology and antibiotic resistance patterns.

The duration of neutropenia and hospitalization according to the underlying disease was longer in the patients with AML compared to those with lymphoid malignancies, with a statistically significant correlation. This is probably due to the disease and treatment-induced myelosuppression being more profound and prolonged in AML because it directly affects granulopoiesis and neutrophils, unlike in ALL and NHL, which primarily involve the lymphoid lineage. Despite this, the duration of neutropenia and hospitalization in both the overall cohort and AML population was shorter compared to literature data, both with and without prophylaxis (Caro et al., 2022), and this could be due to our policy of administering G-CSF during neutropenia.

Our data also showed a high fever rate in the patients who developed severe neutropenia after receiving high-dose CHT. Indeed, our FN rate (80.5%) was higher compared to that reported in similar settings where FQ prophylaxis was used (60–65%) (Cernan et al., 2023; Caro et al., 2022; Averbuch et al., 2013; Legrand et al., 2012), confirming that prophylaxis could reduce FN incidence. However, the FN rate was comparable to that observed in non-prophylaxed patients with AML who received CHT (Douglas et al., 2022; Urbino et al., 2023; Caro et al., 2022). We also observed a higher incidence of fever in the AML group compared to the lymphoid group, probably due to the shorter duration of neutropenia in the lymphoid population. In addition, another possible reason is that fever was masked in the patients with ALL and NHL because of steroid use, which is part of the treatment regimens in these settings. Interestingly, FUO episodes were less common than BSI in our population, which contrasts with what has been reported in the literature (Cernan et al., 2023). This could be due to prophylaxis omission that causes more diagnosed infections, but it could also be a consequence of diagnostic advances made in recent years.

Furthermore, in our cohort, the BSI rate was higher than the prevalence reported in the literature (45.4% in our cohort versus 11–38% in previous studies) (Trecarichi et al., 2023; Urbino et al., 2023; Cernan et al., 2023; Averbuch et al., 2013). This result could be a consequence of the omission of prophylaxis. In fact, recent systematic reviews and observational studies have demonstrated that antibacterial prophylaxis commonly reduces both BSI and bacterial infection rates, although its efficacy is limited in settings with a high prevalence of multidrug-resistant (MDR) bacteria (Singh et al., 2023; Douglas et al., 2022; McClellan et al., 2024). The patients with LNH showed higher BSI rates compared to the patients with ALL and AML. This could be due to both the use of steroids and the typically very high complexity of patients with NHL when treated as inpatients. Indeed, patients with NHL are usually managed as outpatients, and inpatient treatment is chosen mainly for heavily relapsed/refractory cases or very aggressive diseases that require HD CHT. For these reasons, both the prolonged chemotherapy history and the high disease burden complicate their management.

Recent studies have documented a trend reversal and an increasing prevalence of infections caused by Gram-negative bacteria in hematological patients in recent years (Trecarichi et al., 2015; Tumbarello and Raffaelli, 2021; Paul et al., 2010; Wang et al., 2024; McClellan et al., 2024; Kalaskar et al., 2017). This trend was also observed in our population, which showed a higher rate of bacterial infections sustained by Gram-negative bacteria compared to European and local epidemiology [61% in our cohort versus 52.8% described by Trecarichi et al. (2015)]. This may be because FQ prophylaxis, when effective, reduces mainly Gram-negative infections. In line with regional epidemiology, we found that the most frequent BSIs were sustained by gastrointestinal bacteria, as frequently reported in the literature (Trecarichi et al., 2015; Paul et al., 2010; Wang et al., 2024; McClellan et al., 2024; Saini et al., 2013). However, our BSI incidence rate due to MDR bacteria (10.7%) was much lower compared to other reports involving prophylaxis (approximately 31.5–35.2%) (Trecarichi et al., 2023; Urbino et al., 2023; Wang et al., 2024; Averbuch et al., 2013; McClellan et al., 2024), confirming that prior antibiotic exposure could be a potential risk factor for BSI caused by MDR bacteria and that antibiotic use should be revised based on local epidemiology, as noted in previous studies (Wang et al., 2024; McClellan et al., 2024; Tacconelli et al., 2002).

Finally, in our study, the rate of septic shock was 7.6%, which is similar to Urbino et al. (2023) or even lower than the rates reported in the literature (Wang et al., 2024). We did not observe a higher ICU transferring rate or increased IRM compared to the FQ populations, as reported in recent studies (Urbino et al., 2023; Caro et al., 2022).

Omitting FQ prophylaxis could have increased the death rate in our population; however, the OM was 9.3%, and only 7/236 patients died from infections, resulting in an IRM of 3%, similar to, if not better than, comparable settings with or without FQ prophylaxis (Trecarichi et al., 2015; Urbino et al., 2023; Cernan et al., 2023; Neuerburg et al., 2024; Chan et al., 2023; Malagola et al., 2008). This could be due to increasing improvements in FN and BSI management, which progressively reduce mortality despite prophylaxis omission. Regarding the ESBL and CRE colonization rate in the whole cohort of the patients, it was slightly lower compared to the recent literature (Trecarichi et al., 2023). This lower rate could be due to FQ omission, considering that several recent studies have reported an association between antibacterial prophylaxis and subsequent colonization (Singh et al., 2023). A higher, although not statistically significant, rate was observed in the ALL subgroup. This could be partly explained by the severe immunosuppression resulting from humoral immunity deficiency, the extensive use of high-dose steroids, and the characteristic lymphocytopenic effect of CHT treatment in this setting. Notably, omitting FQ prophylaxis in all colonized patients did not lead to an increased incidence of BSI, in contrast to what has been reported in the allogeneic setting (Akhmedov et al., 2023). Indeed, when analyzing BSI epidemiology, we found a higher incidence of Gram-negative bacteria in the patients with colonization (31.8%) compared to patients without colonization (21.8%). However, no statistically significant correlation was observed between non-colonized and ESBL+ or CRE+ patients in terms of FN, FUO, BSI, and mortality, further supporting our policy to omit FQ prophylaxis.

In our cohort, we also analyzed the differences in rates between induction and consolidation treatment cycles. As reported in the literature, especially in patients with acute leukemia (Buckley et al., 2014; Kawasaki et al., 2019), we found a higher incidence of fever during induction therapy. This may be because it usually results in a longer duration of severe neutropenia, increasing the risk of infection. In addition, it is also because in patients undergoing induction CHT, the lysis of a high disease burden may trigger a cytokine storm concurring to cause febrile episodes.

One limitation of our study is the absence of a comparison with a local cohort of similar patients who received FQ prophylaxis. However, we were able to access limited infectious data from 123 patients with AML treated at our center from 2002 to 2014, during which time FQ prophylaxis was routinely administered. In this subgroup of patients, the median duration of neutropenia was 17 days, similar to that of the patients with AML without FQ prophylaxis. The FN episodes were also similar (85% vs. 83.9%), while, as expected, in the earlier FQ group, the incidence of FUO was higher (43.9% vs. 32.4%) and the BSI rate was lower (35.8% vs. 45%).

Another interesting insight could come from the resistance prevalence pattern observed during FQ prophylaxis omission. Unfortunately, we could not collect detailed antibiogram data for each of the 236 HR hematological patients included in this study. However, we were able to compare the overall resistance patterns from 2014, before the cessation of FQ prophylaxis, with those from 2022, 8 years after its discontinuation. Particularly, we observed a reduction in E.coli FQ resistance after the discontinuation of prophylaxis (52% of E.coli FQ resistance in 2014 vs. 32% in 2022), supporting FQ omission and corroborating our policy.

## Conclusion

Although several reports have shown that FQ antibacterial prophylaxis is associated with a lower infection rate and reduced FN, it does not appear to reduce mortality (Hakki et al., 2019; Gafter-Gvili et al., 2012). More recent data (Trecarichi et al., 2023; Mikulska et al., 2018; Tacconelli et al., 2002; Garcia-Vidal et al., 2018), however, suggest that FQ prophylaxis may promote breakthrough infections, with reduced susceptibility to FQ, meropenem, and piperacillin/tazobactam, representing a risk factor for developing BSI sustained by MDR bacteria (Trecarichi et al., 2015). In addition, several studies have underlined that both delayed and inappropriate empiric antibiotic therapy are risk factors for mortality in hematological patients (Barberán et al., 2023; Wang et al., 2024; Garcia-Vidal et al., 2018; Ferrer et al., 2014). Given these premises, in the MDR era, FQ use should be evaluated carefully, considering risks and benefits and avoiding overuse, especially in regions with a high prevalence of resistance. Defining the role of FQ prophylaxis in HR hematological patients has become mandatory.

The results reported here support the decision to safely omit FQ prophylaxis in HR hematological patients, without increasing IRM and while containing the risk of life-threatening infections caused by MDR. However, early diagnosis, close monitoring, prompt antibiotic intervention in cases of FN, and specific training for all practitioners to ensure strict adherence to the infection control protocol are necessary to increase survival rates.

This was a single-center study, and we acknowledge that larger retrospective studies or well-designed prospective trials are needed to confirm our findings and definitively clarify the role of fluoroquinolone (FQ) prophylaxis.

## Data Availability

The raw data supporting the conclusions of this article will be made available by the authors, without undue reservation.
