# Supplementary material for: Low rate of infectious mortality omitting fluoroquinolone prophylaxis in high-risk hematological patients, a single centre experience
Source: Front Microbiol. 2025 Oct 17;16:1632055. doi: 10.3389/fmicb.2025.1632055 (PMC12575246; doi:10.3389/fmicb.2025.1632055)
Supplement: Supplementary file 1 [file Table_1.docx]

| Disease | Treatment regimens | Population |
| --- | --- | --- |
| Acute Myeloid Leukemia | Fludara based ^1,2,3^  3+7 ^4^  CPX351 ^5^  MEC ^6^  HD-ARAC ^7^  other | 94/379 (24,8%)  33/379 (8,7%)  18/379 (4,8%)  38/379 (10,0%)  164/379 (43,3%)  32/379 (8,4%) |
| Acute Lymphoblastic Leukemia | Gimema  NILG 10/07 ^8^ LAL 1913 ^9^ LAL 2317 ^10^  HyperCVAD ^11^  other | 74/90 (82,2%)  9/90 (10,0%)  7/90 (7,8%) |
| Non Hodgkin Lymphoma | HyperCVAD ^11^  CODOX-M-IVAC ^12^  MATRIX ^13^  other | 2/43 (4,7%)  18/43 (41,9%)  13/43 (30,2%)  10/43 (23,2%) |

**Supplementary material.**

1 Parker JE, Pagliuca A, Mijovic A, Cullis JO, Czepulkowski B, Rassam SMB, et al. Fludarabine, cytarabine, G-CSF and idarubicin (FLAG-IDA) for the treatment of poor-risk myelodysplastic syndromes and acute myeloid leukaemia. Br J Haematol. 1997;99(4):939–44. Available from: <https://pubmed.ncbi.nlm.nih.gov/9432047/>

2 Russo D, Malagola M, De Vivo A, Fiacchini M, Martinelli G, Piccaluga PP, et al. Multicentre phase III trial on fludarabine, cytarabine (Ara-C), and idarubicin versus idarubicin, Ara-C and etoposide for induction treatment of younger, newly diagnosed acute myeloid leukaemia patients. Br J Haematol. 2005 Oct;131(2):172–9. Available from: <https://pubmed.ncbi.nlm.nih.gov/16197446/>

3 Russo D, Pricolo G, Michieli M, Michelutti A, Raspadori D, Bertone A, et al. Fludarabine, arabinosyl cytosine and idarubicin (FLAI) for remission induction in poor-risk acute myeloid leukemia. Leuk Lymphoma. 2001 Jan 1;40(3–4):335–43. Available from: <https://pubmed.ncbi.nlm.nih.gov/11426555/>

4 Lichtman MA. A historical perspective on the development of the cytarabine (7days) and daunorubicin (3days) treatment regimen for acute myelogenous leukemia: 2013 the 40th anniversary of 7+3. Blood Cells Mol Dis. 2013 Feb ;50(2):119–30. Available from: https://pubmed.ncbi.nlm.nih.gov/23154039/

5 Lancet JE, Uy GL, Cortes JE, Newell LF, Lin TL, Ritchie EK, et al. CPX-351 (cytarabine and daunorubicin) Liposome for Injection Versus Conventional Cytarabine Plus Daunorubicin in Older Patients With Newly Diagnosed Secondary Acute Myeloid Leukemia. J Clin Oncol. 2018 Sep 10;36(26):2684–92. Available from: https://pubmed.ncbi.nlm.nih.gov/30024784/

6 Amadori S, Arcese W, Isacchi G, Meloni G, Petti MC, Monarca B, et al. Mitoxantrone, etoposide, and intermediate-dose cytarabine: An effective and tolerable regimen for the treatment of refractory acute myeloid leukemia. Journal of Clinical Oncology. 1991;9(7):1210–4.

7 Magina KN, Pregartner G, Zebisch A, Wölfler A, Neumeister P, Greinix HT, et al. Cytarabine dose in the consolidation treatment of AML: a systematic review and meta-analysis. Blood. 2017 Aug 17;130(7):946–8. Available from: <https://pubmed.ncbi.nlm.nih.gov/28679736/>

8 Bassan R, Pavoni C, Intermesoli T, Spinelli O, Tosi M, Audisio E, et al. Updated risk-oriented strategy for acute lymphoblastic leukemia in adult patients 18-65 years: NILG ALL 10/07. Blood Cancer J. 2020 Nov ;10(11). Available from: <https://pubmed.ncbi.nlm.nih.gov/33188164/>

9 Bassan R, Chiaretti S, Starza I Della, Spinelli O, Santoro A, Paoloni F, et al. Pegaspargase-modified risk-oriented program for adult acute lymphoblastic leukemia: results of the GIMEMA LAL1913 trial. Blood Adv. 2023 Aug 16;7(16):4448. Available from: https://pmc.ncbi.nlm.nih.gov/articles/PMC10440455/

10 Chiaretti S, Della Starza I, Santoro A, Spinelli O, Elia L, De Propris MS, et al. Sequential Chemotherapy and Blinatumomab to Improve Minimal Residual Disease in Adult Ph- B-Lineage Acute Lymphoblastic Leukemia. Final Results of the Phase II Gimema LAL2317 Trial. Blood. 2023 Nov 2;142(Supplement 1):826–826. Available from: <https://dx.doi.org/10.1182/blood-2023-174973>

11 Kantarjian H, Thomas D, O’Brien S, Cortes J, Giles F, Jeha S, et al. Long-term follow-up results of hyperfractionated cyclophosphamide, vincristine, doxorubicin, and dexamethasone (Hyper-CVAD), a dose-intensive regimen, in adult acute lymphocytic leukemia. Cancer. 2004 Dec 15;101(12):2788–801. Available from: <https://pubmed.ncbi.nlm.nih.gov/15481055/>

12 Barnes JA, LaCasce AS, Feng Y, Toomey CE, Neuberg D, Michaelson JS, et al. Evaluation of the addition of rituximab to CODOX-M/IVAC for Burkitt’s lymphoma: a retrospective analysis. Ann Oncol. 2011 Aug 1;22(8):1859–64. Available from: <https://pubmed.ncbi.nlm.nih.gov/21339382/>

13 Ferreri AJM, Cwynarski K, Pulczynski E, Ponzoni M, Deckert M, Politi LS, et al. Chemoimmunotherapy with methotrexate, cytarabine, thiotepa, and rituximab (MATRix regimen) in patients with primary CNS lymphoma: results of the first randomisation of the International Extranodal Lymphoma Study Group-32 (IELSG32) phase 2 trial. Lancet Haematol. 2016 May 1;3(5):e217–27. Available from: https://pubmed.ncbi.nlm.nih.gov/27132696/
